# Nutritional intervention and physical training in malnourished frail community-dwelling elderly persons carried out by trained lay “buddies”: study protocol of a randomized controlled trial

**DOI:** 10.1186/1471-2458-13-1232

**Published:** 2013-12-27

**Authors:** Thomas E Dorner, Christian Lackinger, Sandra Haider, Eva Luger, Ali Kapan, Maria Luger, Karin E Schindler

**Affiliations:** 1 Institute of Social Medicine, Centre for Public Health, Medical University of Vienna, Kinderspitalgasse 15/1, 1090 Vienna, Austria; 2 Department for Health Promotion & Prevention, SPORTUNION Austria, Falkestraße 1, 1010 Wien, Austria; 3 Department of Internal Medicine III, Division of Endocrinology and Metabolism, Medical University of Vienna, Waehringer Guertel 18-20, 1090 Wien, Austria; 4 Special Institute for Preventive Cardiology And Nutrition SIPCAN, Guggenbichlerstraße 8/15, 5026 Salzburg, Austria

**Keywords:** Frailty, Community-dwelling, Malnutrition, Physical activity, Strength training

## Abstract

**Background:**

In elderly persons frailty and malnutrition are very common and can lead to serious health hazards such as increased mortality, morbidity, dependency, institutionalization and a reduced quality of life. In Austria, the prevalence of frailty and malnutrition are increasing steadily and are becoming a challenge for our social system. Physical training and adequate nutrition may improve this situation.

**Methods/design:**

In this randomized controlled trial, 80 malnourished frail community-dwelling patients (≥ 65 years) hospitalized at wards for internal medicine are recruited. Additionally, 80 lay volunteers (≥ 50 years), named buddies are recruited and subsequently trained regarding health enhancing physical activity and nutrition in four standardized training sessions. These buddies visit the malnourished frail persons at home twice a week for about one hour during an initial period of 10–12 weeks. While participants allocated to the intervention group (n = 40) receive intervention to improve their fluid intake, protein and energy intake, perform strength training and try to increase their baseline activities, the control group (n = 40) only gets home visits without any intervention. After 10–12 weeks, both, the intervention and the control group, receive the nutritional intervention and the physical training. Health, nutritional and frailty status, physical fitness, body composition and chronic inflammation of buddies and frail persons are recorded before the intervention, after 10–12 weeks, 6 and 12 months.

**Discussion:**

To your knowledge this trial is the first of its kind to provide nutritional and physical activity interventions to malnourished frail community-dwelling persons by trained lay buddies, in which an improvement of the frail persons´ and the buddies’ health status is measured. This study assesses the efficacy of such an intervention and may offer new perspectives for the management of frailty and malnutrition.

**Trail registration:**

ClinicalTrials.gov, NCT01991639

## Background

Frailty is considered as a state of high vulnerability for adverse health outcomes, including disability, dependency, falls, need for long-term care and mortality [1]. Moreover, other difficulties regarding frailty include morbidity, hospitalization, social isolation and an overall decrease in quality of life [2]. According to the Frailty Instrument for Primary Care of the Survey of Health, Ageing and Retirement in Europe (SHARE-FI) [3], the prevalence of frailty in persons older than 65 years is 17%. Additionally, 42.3% are pre-frail, which is an intermediate state between being robust and frail. In Austria, the prevalence of frailty is 10.8%, pre-frailty appears in 40.7% of community-dwelling older people [4]. Through extrapolation of the predicted demographic development, a number of 356,000 frail and 1.5 million pre-frail persons may be expected in 2050 in Austria [5]. Due to this epidemiologic trend, treatment and especially prevention of frailty is becoming one of the greatest challenges for our social system.

Sarcopenia, which is defined as reduced muscle mass and strength and impaired muscle performance [6], significantly contribute to the development of frailty [7]. Underlying reasons for sarcopenia are the ageing process, heritability, an unbalanced diet, a sedentary lifestyle and chronic diseases [8-10]. Within the existing literature, the prevalence of sarcopenia in 60 to 70-year old people is between 5–13% [11] and in the population aged 80 years or older it increases to 11–50% [6].

In addition, malnutrition is associated with a higher risk of becoming frail and it contributes to its pathogenesis [7,12-14]. It is defined as a chronic state in which a combination of over- and undernutrition and inflammatory activity modifies the body composition [15,16] and consequently may lead to serious health hazards [17-22]. According to a review by Guigoz et al. the prevalence of malnutrition in hospitalized patients is 23% and 46% are at risk of malnutrition, respectively [23].

Furthermore, chronic inflammation parameters e.g. leukocytes, interleukin 6 (IL-6), tumour necrosis factor (TNF-alpha) [24,25] and c-reactive-protein (CRP) [24-26] are associated with both, malnutrition and frailty. Moreover, deficits in vitamin D, with its benefit on bone, muscle, and nerve function [27], could be a sign of malnutrition [28,29]. In addition, low levels of serum proteins e.g. albumin and transferrin, and of total cholesterol and triglycerides might indicate malnutrition [30].

As nutritional and frailty status frequently deteriorate post-discharge [31], it can be concluded that energy and especially protein intake must be improved in frail malnourished elderly persons who are still living at home. An individualized nutritional counseling, which takes place three times at patients’ home after discharge, and is conducted by registered dietitian has the potential to improve the nutritional status within 12 weeks [32]. Moreover, exercise training, especially strength training, can improve health status and quality of life [33,34]. According to the fact that a lack of strength is one of the major cause of falls and consequences in frail persons [35-37], strength training may empower elderly people to maintain or regain autonomy and independency. Therefore, a home-based well-structured nutritional intervention program in combination with strength training can be considered as an effective therapeutic option for the treatment of frailty [38] and malnutrition. Due to this intervention the negative outcomes of frailty and malnutrition are expected to be reduced and should help older persons to maintain or even improve their quality of life. Additionally, the general well-being, muscle strength, and activities of daily living are expected to improve [39].

## Methods/design

### Overview

**Figure 1:**
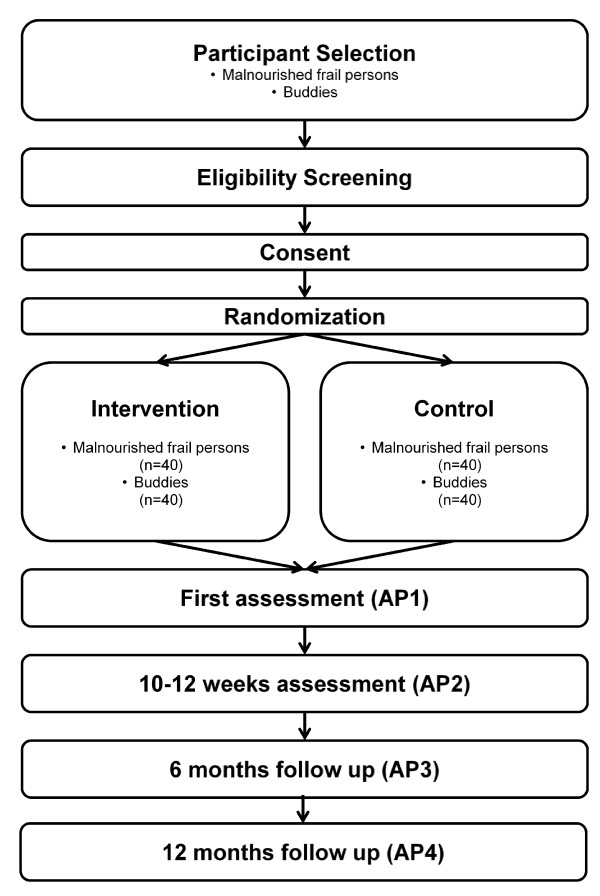
Study design and assessment points.

The proposed study is designed as a prospective randomized controlled trial taking place in Vienna, Austria. A randomization design, which is stratified by handgrip strength, is chosen to get two comparable groups of participants in the intervention and the control group. In the study 80 community-dwelling malnourished frail persons are recruited. They are visited by buddies (volunteers ≥ 50 years) twice a week for about one hour for 6 months. An overview of the study design and the assessment points is provided in Figure 1.

The study was approved by the local ethical committee of the Medical University Vienna (Ref: 1416/2013) and complies with the Declaration of Helsinki [40]. Furthermore, the protocol was registered at clinicaltrials.gov (Identifier: NCT01991639). The study methods are in accordance with the CONSORT guidelines for reporting randomized trials [41].

### Pre-studies

The methods presented in this study are built upon two pre-studies. The findings of the first pre-study illustrated that 54.13% of people over the age of 65 years (n = 133) inpatient in the ward for internal medicine in acute hospitals in Vienna are frail and 25.56% are malnourished. Additionally, even more are at higher risk (pre-frail: 21.8%; at risk of malnutrition: 51.13%) [39,42]. Moreover, this pre-study showed that 64.7% of the participants are willing to improve their health situation and they are interested in a home-based program (64.7%), which is based on strength training and improvement of nutritional habits.

The second pre-study by Müller et al. proved that with the help of the Austrian charitable organizations it is possible to recruit a sufficient number of volunteers, who are willing to perform and supervise the exercise program and the nutritional intervention. Furthermore, in this pre-study the buddies were trained and educated in a one-day workshop. After one month their knowledge and skills concerning the most important aspects of healthy nutrition and exercise training were tested. The results of this pilot study showed that with an extensive preparation lay people, older than 50 years, are certainly capable to conduct these interventions [43]. However, a several-day workshop should be preferred to train the buddies.

### Study objectives

The primary objective of the study is to increase the handgrip strength by this intervention, measured with a dynamometer. Further aims of the proposed study are to improve the nutritional status, to increase the amount of health enhancing physical activity and to improve the overall health status in malnourished frail community-dwelling older people and buddies.

Due to the project design, the social networks and social interaction of buddies and frail person is expected to be strengthened. In addition, health resources and quality of life should be increased.

A clear objective of this study is to perform the nutritional and physical training intervention in malnourished frail people’s home environment.

### Recruitment and eligibility

#### Malnourished frail persons

Malnourished frail people 65 years and older, who are inpatient in five hospitals in Vienna, wards for internal medicine, and close to discharge are recruited in four waves within one year. The inclusion and exclusion criteria for malnourished frail persons are shown in Table 1.

**Table 1:** Inclusion and exclusion criteria of frail malnourished persons

**Inclusion criteria**	**Exclusion criteria**
▪65 years or older	▪Planned admission to nursing home
▪Resident in Vienna	▪Chemo or radiotherapy at the moment or planned
▪Malnutrition or at risk of malnutrition according to the MNA-SF (≤ 11 points) OR	▪Nursing level 6^a^ or 7^b^
Frail or pre-frail according to the SHARE-FI (female: >0.315; male: >1.212 points)	
▪Community-dwelling	▪Insulin treated diabetes mellitus according to the medical charts
▪No medical contraindication for the performance of strength training (“No” to the question ‘Has your doctor recently told you that you should not exercise?”)	▪COPD stage III or IV
▪Able to walk (with or without a walking aid)	▪Dialysis patient or chronic kidney insufficiency with protein restriction
▪Capability to consent	▪Cannot understand the German language
	▪Impaired cognitive function according to the MMSE (≤ 17 points)

^a^more than 180 hours of care is necessary; care cannot be planned or is permanently required.

^b^more than 180 hours of care is necessary; person cannot move without help.

#### Buddies

The buddies are recruited in cooperation with the “Wiener Hilfswerk” in four waves within one year. The “Wiener Hilfswerk” is one of the largest organizations in Austria, which already offers care-giving services for elderly persons carried out by volunteers. The inclusion criteria for buddies are:

• 50 years or older

• Readiness to participate in the study either in the intervention or control group

• Commitment to keep to the protocol for at least 6 months

### Sample size calculation

For sample size calculation, the difference in handgrip strength from the first assessment point (AP1) to the second assessment point (AP2), which take place 10–12 weeks after AP1 (see Figure 1), is considered as a marker of muscle strength. We examine its relationship with diet [24,33] and physical activity [34] in a community-dwelling cohort. Given a clinically relevant difference of 2 kg in handgrip strength (intervention group improves by 2 kg more than the control group in handgrip strength), a standard deviation of 3 kg of the differences, a two-sided significance level of 0.05, a sample size of n = 36 per group is needed to reach 80% statistical power. Since imputation for drop-outs may have some inestimable effect on the assumed standard deviation of the differences, the sample size is increased to n = 40 per group. Expected value μ1 (intervention) = 16 kg, μ2 (control) = 14 kg (based on the results of the pre-study [42,44]). The primary endpoint is analyzed according to the intention-to-treat principle (ITT).

### Randomization

Persons are randomly assigned to the intervention or control group, stratified by handgrip strength with the help of the “Randomizer for Clinical Trials 1.8.1″ [45]. Randomization is carried out after the patient has signed the informed consent and has been matched to a lay buddy, dependent on the place of residence.

### Intervention

In the first 10–12 weeks each couple (one buddy and one malnourished frail person) is divided randomly into the intervention and the control group.

### Control group

Participants in the control group are visited twice a week by buddies, but they do not specifically monitor the nutritional status or perform physical training in the first 10–12 weeks. Instead of that, buddies are provided with a portfolio of possible activities, especially cognitive training, which they could perform together with the frail malnourished person. After 10–12 weeks the control group also receives nutritional intervention and the physical training for 3 months.

### Intervention group

Buddies visit malnourished frail older persons twice a week for approximately one hour and they perform nutritional and physical activity interventions.

#### Activities for improving nutritional habit

The aim is to obtain adequate protein, energy and other nutrient intake, preferably by regular foods and beverages. Therefore, buddies discuss the following three main nutritional messages with the malnourished frail persons: fluid intake**,** animal and plant protein intake and energy intake. For this purpose, buddies are equipped with a portfolio in which the topics concerning physical activity and nutrition are explained in a straightforward manner. Moreover, they obtain ideas how to enrich food with protein and receive recipes of dishes which are protein and energy dense.

**Figure 2:**
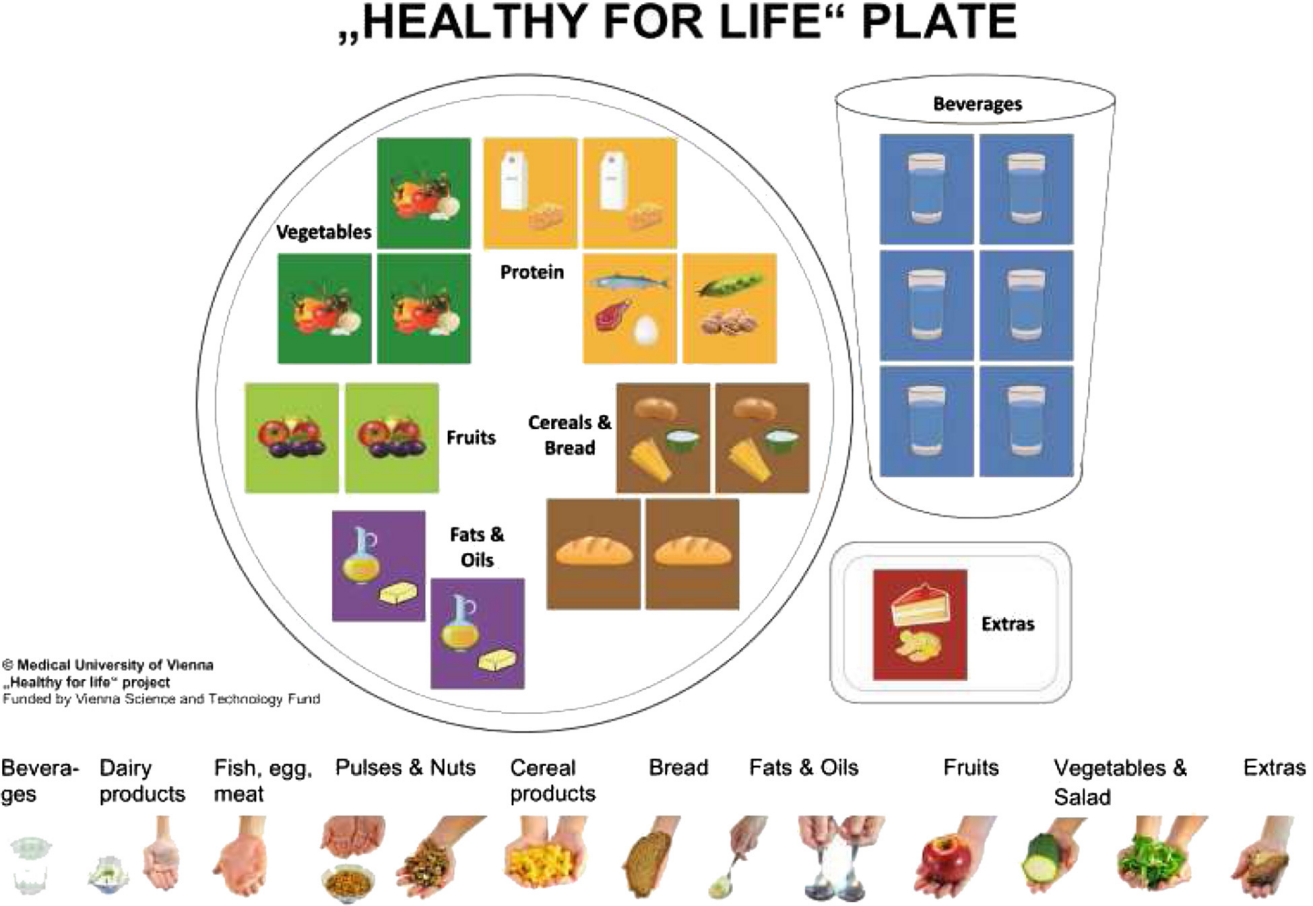
Healthy for life plate.

In order to show the variance of recommended and actual food intake, buddies are equipped with the “Healthy for Life Plate” which is a modification of the Health Eating Plate of the Harvard University [46]. It consists of a play board and food-cards representing a variety of foods (Figure 2).

One food-card shows one portion size. Buddies and malnourished frail persons should set these food-cards, which they have eaten the day before, on the play board. The “Healthy for Life Plate” assesses the quantity and the composition of daily food rations. Therefore, with the game, dietary behavior can be examined and consequently optimize dietary habits. For convincing malnourished frail persons to change their nutritional habits, buddies additionally use motivational interviewing techniques [47]. Due to this fact every nutritional message includes a section for individual goal setting and tools to reinforce the self-efficacy. As it is done in the motivational-volitions-concept [48], every message also includes a part which should reveal problems of implementing the goal in everyday life, in order to find solutions for them.

#### Physical intervention

Twice a week strength training is performed by malnourished frail persons together with the buddies. The training comprises a warm-up (about five minutes, mobilization exercises) followed by six strength exercises, which are performed in circuit form with two sets. The exercises are conducted with 15 repetitions until muscular exhaustion. The performed exercises can be retained from Table 2. This circle lasts about 30 minutes. Moreover, buddies talk about the relevance of health enhancing physical activity in connection with frailty. Further on, they discuss possible arising problems, talk about their baseline activity and set individual goals concerning physical activity. For personal motivation buddies and frail people get a pedometer. Additionally, buddies advise the malnourished frail elderly to practice the same strength exercises once a week on their own.

**Table 2:** Description of the six strength exercises

**Basic exercise**	**Alternative exercise**	**Muscle group**
▪Mini squat in front of a chair (with the help of a table)	▪Lunges	▪Femoral muscles
▪Chest Press against elastic resistance - sitting on a chair	▪Chest Press against the wall	▪Pectoral muscles
▪“beetles” - sitting on the chair		▪Abdominal muscles
▪Hip extension - standing position	▪Hip extension - standing position against elastic resistance	▪Ischiocrural muscles
▪Reverse Butterfly against elastic resistance - sitting on a chair		▪Upper back muscles
▪Shoulder press against elastic resistance - sitting on a chair		▪Muscles of the arms, and shoulders

#### Materials for the intervention group

Participants of the intervention group receive a bag which contains the following materials: a guidebook including messages regarding health promoting nutrition, detailed information on health enhancing physical activity and all strength exercises shown as pictures, a recipe book with dishes high in energy and protein, a dynaband for exercising, a demonstration DVD for guidance and motivation and a pedometer for counting food steps. Additionally, in the box there is the “Healthy for Life Plate”. Buddies are also equipped with a documentation book, where they should record the content of each home visit.

### Training of the buddies

**Figure 3:**
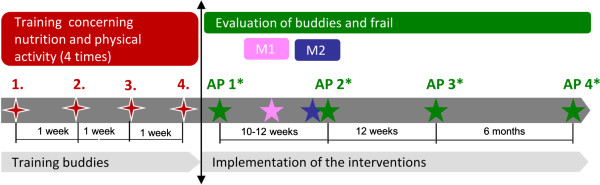
**Training of buddies.** AP: Assessment point. M1: Meeting to discuss open questions from the buddies concerning the interventions. M2: corrective training for the control group.

The project team, consisting of three sport scientists, two nutritional scientists and one medical doctor, trains the buddies of the intervention and the control group 4 times for 3 hours each session. This training starts 4 weeks before the first appointment with the malnourished frail persons will take place (Figure 3).

The sessions comprise lessons concerning aging, frailty, malnutrition, main nutritional messages, which are described in the section intervention group, and the importance of health enhancing physical activity, focusing on strength training and baseline activity. Moreover, buddies learn about the key issues of motivational interviewing skills. Buddies additionally get to know the interventions conducted with the control group. Training is designed interactively and enables buddies how to enrich dishes with regards to the energy and protein intake. They practice the motivational interviewing skills and can taste food. Furthermore, the project team exercises together with buddies in order to show them the right performance and intensity of each strength exercise and they discuss their pedometer data with them.

Two to three weeks after the beginning of the intervention, the buddies and the project team meet again to discuss arising questions and exchange experiences.

Buddies in the control group get another training to refresh their knowledge before they start with the nutritional and physical activity intervention after 10–12 weeks of the initial period.

### Measurements

All involved persons, malnourished frail elderly people and buddies, are evaluated at four points in time: before the intervention (AP1), after 10–12 weeks (AP2), after 6 months (AP3), and after 12 months (AP4). Malnourished frail persons are measured at their home and all buddies are surveyed after the last training of the buddies, respectively. Not all measurements take place at each time point. Table 3 gives a summary of the conducted measurements at several times (AP1, AP2, AP3, AP4) in malnourished frail elderly persons (F) and buddies (B).

**Table 3:** Measurements at several timepoints in malnourished frail elderly persons (F) and buddies (B)

	**Selection**	**AP1 (baseline)**		**AP2 (after 12 weeks)**		**AP3 (after 6 months)**		AP4* (after 12 months)	
	**F**	**F**	**B**	**F**	**B**	**F**	**B**	**F**	**B**
Long-term medication	X								
Comorbidities	X								
WHOQOL		X	X	X	X	X	X		
WHOQOL-OLD		X		X		X			
F-SozU		X	X	X	X	X	X		
Frequency of hospitalization		X		X		X			
Frequency of falls		X		X		X			
FES-I		X		X		X			
MNA-SF	X								
MNA-LF		X		X		X		X	
SHARE-FI	X			X		X		X	
MMSE	X			X		X		X	
BIA		X	X	X	X	X	X	X	X
Anthropometry		X	X	X	X	X	X	X	X
MEDIAS		X	X	X	X	X	X		
Food frequency questionnaire (Proteins)		X	X	X	X	X	X		
Supplements		X	X	X	X	X	X		
Protocol & picture of the refrigerator content		X		X		X			
MASS		X	X	X	X	X	X		
Concept 2 Dyno			X		X		X		
Pedometer			X	X	X	X	X		
Modified PASE		X	X	X	X	X	X		
Modified FEG			X		X		X		
SPPB		X		X		X			
Laboratory parameters		X	X	X	X	X	X		
Personal information		X	X						
Drop-out				X	X	X	X	X	X
Adherence				X	X	X	X	X	X
Expectations/appraisal of the intervention		X	X	X	X	X	X	X	X

WHOQOL = World Health Organization Quality of Life; F-SozU = Fragebogen zur sozialen Unterstützung; FES-I = Falls Efficacy Scale International; MNA-SF = Mini nutritional assessment Short-Form; MNA-LF = Mini nutritional assessment Long-Form; SHARE-FI = Frailty Instrument for Primary Care of the Survey of Health, Ageing and Retirement in Europe; MMSE = Mini-Mental State Examination; BIA = Bioelectrical Impedance Analysis; MEDIAS = Mediterranean Diet Adherence Screener; MASS = Measurement of age and sex related reference values of muscle strength; PASE = Physical Activity Scale for the Elderly; FEG = Fragebogen zur Erfassung des Gesundheitsverhaltens; SPPB = Short Physical Performance Battery.

*AP4 is voluntary.

The following measurements are performed:

Measurements of health status:

• *Quality of life* assessed by the World Health Organization Quality of Life WHOQOL-BREF [49] and by 3 dimensions of the WHOQOL-OLD (“sensory functions”, “autonomy”, “activities in the past, present and future”) [50]

• *Social support* measured with the short version of the questionnaire F-SozU [51].

• *Frequency of hospitalization*

• *Frequency of falls*

• *Documentation of fear of falling* assessed by the FES-I (Falls Efficacy Scale-International Version) [52]

Measurements of frailty, nutritional status, physical activity:

• *Frailty status* assessed by the SHARE-FI (Frailty Instrument for Primary Care of the Survey of Health, Ageing and Retirement in Europe) [3]. According to the results of the SHARE-FI, persons are categorized in frail, pre-frail, and robust people divided by gender. The handgrip strength, which is the primary endpoint of the study and part of the SHARE-FI, is measured with a hydraulic dynamometer in standardized procedure [53]. For each side three attempts are made, and the highest one is used for calculation.

• *Nutritional status* assessed by the long form of the MNA (Mini Nutritional Assessment) [54]. In our study calf circumference is used which is measured with a tape at the sitting patient on the left and the right lower leg at the strongest circumference [55]. Different to the protocol, persons put their feet on the floor while measuring.

• *Cognitive function* assessed by the German version [56] of the MMSE (Mini Mental State Examination) [57].

• *Body mass index (BMI; kg/m*^*2*^*)* while body height is assessed by a yardstick and body weight by a calibrated scale.

• *Body composition* assessed by the BIA (Bioelectrical Impedance Analysis) in standardized procedure [58]. Moreover, waist circumference is measured at a vertical level 2.5 cm above the umbilicus while expiration [59].

• *Quality of the diet* assessed by the Mediterranean Diet Adherence Screener (MEDIAS) [60], a food frequency questionnaire assessing the protein intake and questions concerning supplements [61].

• *Qualitative and quantitative contents of refrigerators* assessed by a predefined protocol and picture of refrigerator [62].

• *Muscle strength* assessed by the MASS (Measurement of age and sex related reference values of muscle strength) and the Concept 2 Dyno. The MASS is a new diagnosis system which was developed by the Technical University of Vienna. It evaluates health related concentric dynamic muscle strength. The measurement is velocity-independent and hence, is appropriate for the target group. Three exercises (bench press, bench pull, and leg press) will be conducted in standardized procedure. For all tested muscle groups the following parameters are recorded: maximum resistance with low velocity and 2–3 fix adjusted sub-maximum loads. Moreover, the Concept 2 Dyno is used for assessing the muscle strength. The same exercises as before will be conducted in standardized procedure [63].

• *Daily physical activity* assessed by an activity sensor (pedometer), the modified PASE (Physical Activity Scale for the elderly) [64] and the modified questionnaire on health-related behavior FEG (Fragebogen zur Erfassung des Gesundheitsverhaltens) [65]. The pedometer-data will be analyzed as follows: Data from the week before AP2, AP3 and AP4 will be included in the analysis. Data of those who have used the pedometer less than four days are excluded. Days, on which the pedometer was used less than eight hours, are considered as invalid. The number of steps below 90 steps per minute, the number of steps above 90 steps per minutes and the walk time is analyzed.

• *Balance and mobility components* assessed by the SPPB (Short Physical Performance Battery) [66].

• *Laboratory parameters* for malnutrition or frailty (albumin, total cholesterol, transferrin, triglyceride, 25-hydroxy-vitamin D, folic acid, CRP, IL-6, TNF-alpha and leucocytes)

Other measurements

• *Personal data* e.g. age, gender, family status, education level, income, occupation

• *Measures of drop-out (DO)*[67]*:* the drop-out rate between the first and second visit and until the final visit should be analyzed. For each case of discontinued participation (Ri), the following reasons are discriminated: medical reason (R1) and person’s own decision (R2), whereas an “A” stands for all participants who passed the baseline assessment.

• Drop-outs between the first and second visit:

⋅DO_R_ = DO_1_ + DO_2_ – all participants who drop out between the first and second visit

⋅DO_R1_ = DO_1_/A – percentage of participants who discontinue by medical reasons.

⋅DO_R2_ = DO_2_/A – percentage of participants who discontinue on their own decision. Members of this group are asked for a personal reason (voluntary and open question), and also be asked to participate in the final visit.

• Drop-outs until the final visit

⋅|DOR| – total number of participants who drop out until the final visit

⋅IDORI = IDO1I + IDO2I – all participants who drop out until the final visit

⋅IDOR1I = IDO1I/A – percentage of participants who discontinue by medical reasons.

⋅IDOR2I = IDO2I/A – percentage of participants who discontinue on their own decision.

• *Measures of adherence* are calculated as:

⋅Number of home visits

⋅Number of activity units, which have been done without buddies respectively without malnourished frail people

⋅Number of discussed nutritional messages

• *Expectations/Appraisal of the interventions by participant (malnourished frail persons and buddies)*[67]*:* following questions are asked, “Would you once again participate in the trial? Why/why not?”. Participants, who completed the intervention, are asked to rate the content of the nutritional and physical activity interventions, the length of the program, and the frequency of home visits. Moreover, they should score (on a scale from one to five) the utility of the materials, which they received e.g. guidebook, dynabands, pedometers, training DVD or VHS, the “Healthy for Life Game” and questionnaires. Buddies should also rate the training sessions and the documentation book. Finally, concrete proposals for program improvements are asked, “Do you have any ideas or proposals for program improvements?”.

• *Documentation of any undesirable event during the intervention:* Any symptom or any disease of a participant, which occurs during the intervention, is called an undesirable event [67]. This definition is valid, whether this event is caused by the intervention or not. In case of an undesirable event, the participant promptly has to suspend nutritional and exercise units and they have to visit a physician. The event has to be documented by a standardized report protocol, including the following judgments: medical/non-medical, caused/not caused by exercises and people may/may not continue nutritional and exercise units.

### Statistical analysis

Data exploration using descriptive statistical analysis and inferential statistics is performed. The sample data is carried out by frequencies or percentages, means and standard deviation, and graphics. 95% confidence intervals (CI) are calculated for the differences in percentages and medians. T-test and chi-square test is used to compare groups at baseline. Moreover, Pearson’s- and Spearman’s correlation coefficients is used. In order to test the normal distribution, histograms and box plots will be applied. If normal distributions are not met, non-parametric tests such as Wilcoxon, Mann–Whitney-U-Test and Kendall’s tau and Spearman’s correlation coefficients are chosen. Analysis of covariance (ANCOVA), comparing parameters after the intervention (AP2) and after the follow-ups (AP3, AP4) between intervention and control group, adjusting for the baseline value as covariate is performed. The IBM® SPSS® Statistics for Windows, Version 20 software (IBM Corp., Armonk, NY, U.S.) is used for all statistical analyses. All tests are two-sided and a p-value <0.05 is considered statistically significant.

## Discussion

The major strength of the proposed study design is the implementation of nutritional and physical activity interventions by trained lay buddies on malnourished frail people at their home environment. The results of previous studies on supervised strength training in frail persons demonstrate beneficial effects on risk of falls, balance or on gait ability [68-71]. Additionally, other studies examined the effects on home-based exercise programs supervised by therapists or home helpers [72-76]. Especially, *Vestergaard S* and colleagues showed in a randomized study a significant improvement of handgrip strength in community-dwelling frail older women, implemented by home-based video exercise training interventions [77]. Furthermore, the randomized study by Bonnefoy M et al. demonstrates that the compliance rate of people who were supervised by people with lower qualification levels is not much lower than those who obtained an intervention by specially-trained nurses [74]. Moreover, there are indications in the literature that the nutritional status is getting better after nursing home admission [78] and that an individualized nutritional counseling can improve their dietary habit of community-dwelling elderly person after discharge [32]. On basis of these findings we are convinced that trained lay buddies are able to improve the health status of malnourished frail persons. An additional advantage of our study design is the need for malnourished frail people to leave their home, which might be an impeding factor for participating in such interventions. Furthermore, this designed study has the potential to improve the overall health status of buddies. Within the scope of the study, buddies experience the capability to prevent frailty and malnutrition by a balanced dietary pattern and physical activity. A reduction of isolation should be reached in both concerned parties.

A crucial point of the study is the compliance of both parties on regularly performing the strength exercises with correct intensity. Hence, all four buddy training sessions focus on this issue. Another crucial point is the ability of buddies to motivate frail malnourished persons to improve their nutritional habits. As the buddies are non-professionals, the nutritional interventions focus on the main “topics”. In detail, these topics contain information and recommendations on nutrition and additionally suggestions on the implementation in everyday life e.g. modified traditional recipes with tips on protein enrichment or portion sizes shown as handful in pictures.

The main objectives of these nutritional and physical activity interventions are the evaluation of the applicability of this program carried out by buddies and especially the sustainability that older people may live independently at home and as long as possible, which offers new methods for the management of frailty.

## Abbreviations

AP1: Assessment point1; AP2: Assessment point2; AP3: Assessment point3; AP4: Assessment point4; B: Buddies; BIA: Bioelectrical impedance analysis; F: Malnourished, frail, older persons; FEG: Fragebogen zur Erfassung des Gesundheitsverhaltens; FES-I: Falls Efficacy Scale-International Version; F-SozU: Fragebogen zur sozialen Unterstützung; MASS: Measurement of age and sex related reference values of muscle strength; MEDIAS: Mediterranean diet adherence screener; MMSE: Mini mental state examination; MNA: Mini nutritional assessment; MNA-LF: Mini nutritional assessment long-form; MNA-SF: Mini nutritional assessment Short-Form; PASE: Physical activity scale for the elderly; SHARE-FI: Frailty instrument for primary care of the survey of health, ageing and retirement in Europe; SPPB: Short physical performance battery; WHOQOL: World Health Organization Quality of Life.

## Competing interests

The project has been funded by grants from the WWTF (LS12-039). The authors declare that they have no competing interests.

## Authors’ contributions

TED is the principle investigator of the study, designed the study together with CL and KES, prepared the grant application and drafted the manuscript. EL, SH and AK are responsible for the elaboration and realization of the project. ML advised on editing and corrected the manuscript. All authors have read and approved the final version of the manuscript.

## Authors’ information

TED: medical doctor, associate professor at the Institute of Social Medicine, Centre for Public Health, Medical University of Vienna.

CL: sports scientist, SPORTUNION Austria, Department for Preventive Medicine.

SH: sports scientist and PHD-student at the Institute of Social Medicine, Centre for Public Health, Medical University of Vienna; sports scientist, SPORTUNION Austria, Department for Preventive Medicine.

EL: nutritionist and PHD-student at the Institute of Social Medicine, Centre for Public Health, Medical University of Vienna.

AK: sports scientist and PHD-student at the Institute of Social Medicine, Centre for Public Health, Medical University of Vienna.

ML: nutritionist at the Department of Internal Medicine III, Division of Endocrinology and Metabolism, Medical University of Vienna and at the Special Institute for Preventive Cardiology And Nutrition SIPCAN.

KES: nutritionist at the Department of Internal Medicine III, Division of Endocrinology and Metabolism, Medical University of Vienna.

## Pre-publication history

The pre-publication history for this paper can be accessed here:

http://www.biomedcentral.com/1471-2458/13/1232/prepub
